# Verification of the Self-Healing Ability of PP-co-HUPy Copolymers in Epoxy Systems

**DOI:** 10.3390/polym16111509

**Published:** 2024-05-27

**Authors:** Elisa Calabrese, Marialuigia Raimondo, Andrea Sorrentino, Simona Russo, Pasquale Longo, Annaluisa Mariconda, Raffaele Longo, Liberata Guadagno

**Affiliations:** 1 Department of Industrial Engineering, University of Salerno, Via Giovanni Paolo II, 132, 84084 Fisciano, Italy; elicalabrese@unisa.it (E.C.); rlongo@unisa.it (R.L.); 2 Institute of Polymers, Composites and Biomaterials (IPCB-CNR), via Previati n. 1/E, 23900 Lecco, Italy; andrea.sorrentino@cnr.it; 3 Department of Chemistry and Biology, University of Salerno, Via Giovanni Paolo II, 132, 84084 Fisciano, Italy; sirusso@unisa.it (S.R.); plongo@unisa.it (P.L.); 4 Department of Science, University of Basilicata, Viale dell’Ateneo Lucano, 10, 85100 Potenza, Italy; annaluisa.mariconda@unibas.it

**Keywords:** PP-co-HUPy copolymers, thermosetting resins, supramolecular interactions

## Abstract

This work concerns the verification of the self-healing ability of PP-co-HUPy copolymers dispersed in epoxy systems. PP is the acronym for the Poly-PEGMA polymer, and HUPy refers to the HEMA-UPy copolymers based on ureidopyrimidinone (UPy) moieties. In particular, this work aims to verify whether this elastomer characterized by an intrinsic self-healing ability can activate supramolecular interactions among polymer chains of an epoxy resin, as in the elastomer alone. The elastomer includes a class of polyethylene glycol monomethyl ether methacrylate-based copolymers, with different percentages of urea-N-2-amino-4-hydroxy-6-methyl pyrimidine-N’-(hexamethylene-n-carboxyethyl methacrylate) (HEMA-UPy) co-monomers. The self-healing capability of these copolymers based on possible quadruple hydrogen bond interactions between polymer chains has been verified. The formulated epoxy samples did not show self-healing efficiency. This can be attributed to the formation of phase segregation that originates during the curing process of the samples, although the PP-co-HUPy copolymers are completely soluble in the liquid epoxy matrix EP. The morphological investigation highlighted the presence of crystals of PP-co-HUPy copolymers, which are in greater quantity in the sample containing the highest weight percentage (7.8 wt%) of HUPy units. Furthermore, the crystals act as promotors for increasing the curing degree (DC) of the epoxy systems containing HUPy units. DC goes from 91.6% for EP to 96.1% and 95.4% for the samples containing weight percentages of 2.5 and 7.8 wt% of HUPy units, respectively. Dynamic mechanical analysis (DMA) shows storage modulus values for epoxy systems containing PP-co-HUPy units lower than that of the unfilled resin EP. The values of maximum in Tan δ (Tg), representing the temperature at which the glass transition occurs, are 220 for the unfilled resin EP, 228 for the sample containing 2.5 wt% of HEMA-UPy units, and 211 for the sample containing 7.8 wt% of HEMA-UPy units.

## 1. Introduction

The fuel economy is of great interest in aviation and is the determining aspect of developing new materials. Being lightweight, therefore, is a crucial aspect. The use of lightweight materials enables lower fuel consumption (for the same route), leading to lower costs, good environmental sustainability, and the maximization of vehicle performance. For these reasons, in recent decades, composite materials have increasingly replaced heavier metal alloys in structural applications. 

Special attention is now paid to the long-term durability of the materials designed for structural applications, especially in aviation [1,2,3,4,5,6]. 

The impact of hail on fuselages during a storm, the impact of stones during landing, and that of birds in flight can lead to micro-cracking of the matrices that can extend over time, catastrophically damaging the materials and reducing their lifetimes [7,8,9,10,11,12]. 

From this perspective, it is pioneering for the aviation industry to make composite materials with self-healing abilities to overcome damage diagnosis and repair difficulties, reduce maintenance costs, and simultaneously cut down the amount of waste, with a view to sustainability. 

Self-healing systems are smart materials as they can extend a material’s shelf-life by independently triggering repair processes: they are designed to repair microcracks and stop their extension, which would lead to more significant damage to the material [13,14,15,16,17]. 

Recently, this class of materials has caught on in many areas, such as the automotive and construction industries. In this context, paints capable of auto-repairing scratches and surface damage are highly representative [18,19,20,21]. 

Depending on the approaches investigated over the years to integrate self-restoring abilities in several matrices, self-healing systems can be classified as extrinsic or intrinsic [22,23,24,25,26,27].

In extrinsic systems, the healing agent has to be pre-incorporated into the desired matrix: damage recovery occurs through the polymerization of a microencapsulated healing agent that is released in conjunction with the rupture event and reacted with a suitable catalyst, both dispersed within the matrix of interest [28,29,30]. 

On the other hand, intrinsic self-healing agents can repair the microcracks by themselves [31].

Our research group has been working on the realization of self-healing polymer composites for many years and, inspired by White’s system [32], which is the first extrinsic system reported in the literature, we realized a similar system for aeronautical application by synthesizing a new stable ruthenium catalyst capable of overcoming the critical points related to the temperature necessary in the processing of industrial resins and the aircraft’s operating temperature [33,34].

However, the main disadvantage of this kind of system is related to the cost of the ruthenium catalyst and the impossibility of healing multiple times at the same site [35].

For this reason, the scientific community has been working for years on replacing these systems with intrinsic ones based on reversible and movable crosslinks, such as hydrogen bonds, ionic interactions, π–π stacking interactions, metal coordination bonds, host–guest interactions, and dynamic covalent bonds (such as the Diels Alder cycloaddition reaction), which allow multiple repair cycles at the same site [36,37,38,39,40,41,42,43,44].

Cross-linking between polymer chains due to hydrogen bond-type interactions is one of the simplest strategies for making self-repairing materials due to its directionality and versatility [45]. 

A milestone in the field of self-healing by hydrogen bonding interaction is the work of Beijer et al., which describes the synthesis of the ureidopyrimidinone (UPy) dimer considered “the novel building block for self-assembly”, due to its high tendency to dimerize by quadruple hydrogen bond interactions [46,47].

In the following years, a great deal of papers focused on the realization of polymers containing UPy units [48].

In particular, Chen et al. realized a polymer Poly(PEGMA-co-UPy) by incorporating the same UPy moieties into poly(ethylene glycol) methyl ether methacrylate (PEGMA), via free radical polymerization of PEGMA with Urea-N-2-amino-4-hydroxy-6-methylpyrimidine-N′-(hexametylen-N-carboxyethyl methacrylate) (HEMA-UPy). The in situ polymerization of pyrrole within the so-formed polymer matrix has resulted in a self-healing, polypyrrole-doped, conductive, multifunctional material for electronic sensors [49].

Recently, we reported a slightly modified synthesis procedure of the same supramolecular elastomer Poly(PEGMA-co-UPy) based on ureidopyrimidinone moieties, in different compositions of the monomer PEGMA and co-monomer HEMA-UPy, and its self-healing ability was evaluated by thermomechanical analysis. 

In particular, an innovative method based on dynamic mechanical analysis (DMA) investigation has been proposed to obtain information about the healing efficiency of the Poly(PEGMA-5-UPy) copolymer at different values of temperatures and frequencies [50]. 

In 2021, Zhang and co-workers described the preparation of a self-healable UPy-modified epoxy resin defined SH-E51-x that was obtained by adding a UPy-based supramolecular polymer (300 Nsp) and the curing agent (D1000) into the modified bisphenol-A glycidyl ether (UPy-E51), whereas the meaning of x in SH-E51-x stays for the mass fractions of the 300 Nsp polymer.

The E51 resin has been functionalized with UPy groups to improve its compatibility with Nsp polymer ending with the same groups: in this way, the UPy moieties on the side chain of the modified UPy-E51 resin can interact with the UPy groups of the supramolecular polymer thanks to the hydrogen bonds.

They found that after heating for 2 h at 90 °C, the SH-E51-x resins could be healed by more than 95% [51,52].

With this contribution, starting from the interesting result of our last work, we have investigated the self-healing ability of the composite material resulting from the combination of 5% by weight of the copolymers Poly(PEGMA-2.5-UPy) and Poly(PEGMA-7.8-UPy) with the epoxy resin that has been chosen for aeronautical applications.

The selected matrix, here referred to by the acronym EP, is an aeronautical resin obtained by mixing the tetraglycidyl methylene dianiline, a tetrafunctional epoxy precursor, with 4,4-diaminodiphenyl sulfone, which is the curing agent, and 1,4-Butanediol-diglycidylether, which is the reactive diluent [8].

Unlike Zhang and co-workers, we did not functionalize the EP resin to improve its interaction with the Poly(PEGMA-co-UPy) elastomer because it is extremely important in aviation to preserve the resin’s mechanical properties as much as possible. 

With this work, we want to show that the establishment of hydrogen bond-type interactions between the pristine resin and the polymer chains is enough to ensure good dispersion. The hydrogen bond-type interaction, therefore, is central to this work because it is both responsible for the self-healing capabilities of the resulting composite material and for obtaining a homogeneous system since this kind of interaction is supposed to exist not only between the polymer chains but also between the polymer chains and the resin. In particular, we want to verify the self-healing capability of these copolymers based on possible quadruple hydrogen bond interactions between polymer chains. 

Thermogravimetric analysis (TGA), differential scanning calorimetry (DSC), Fourier transform infrared spectroscopy (FTIR) measurements, and Field emission scanning electron microscopy (FESEM) have been used to verify this hypothesis and show how the hydrogen-bond interactions affect the thermal stability. 

In addition, dynamic mechanical analyses (DMA) were conducted to evaluate the samples’ mechanical properties and self-healing ability. In this regard, we have demonstrated that, due to the phase segregation that arises when epoxy systems loaded with different quantities of PP-co-HUPy units are subjected to a high-temperature curing process up to 200 °C, these copolymers are not capable of activating quadruple hydrogen bond interactions between polymer chains, preventing the self-repair of the final material.

This study demonstrates that even in the presence of strong hydrogen bond interactions, phase segregation of PP-co-HUPy units inside the epoxy matrix during the curing process can alter local material properties, such as hardness, resilience, and local chemical composition. This also determines an inefficacy in transferring the self-healing ability to the hosting epoxy matrix despite the excellent miscibility of the elastomer with the matrix components in the fluid phase. Furthermore, units of PP-co-HUPy accelerate the curing degree (DC) of the resin system. The phase segregation leads to reduced cohesion between different phases of the material, weakening the interface and making the material more susceptible to fracture. The phase segregation also determines a decrease in the storage modulus of the formulated epoxy systems. In contrast, it does not significantly compromise the value of tan δ, which essentially depends almost exclusively on the epoxy matrix. 

This paper aims to contribute to understanding the mechanisms linked to the combination of different components and how these can impact the microstructure of the final materials, influencing their final performance.

## 2. Materials and Methods

### 2.1. Materials 

Detailed information regarding all the reagents used to synthesize PP-co-HUPy copolymers (PP is the acronym for the Poly-PEGMA polymer, where PEGMA is polyethylene glycol monomethyl ether methacrylate, and HUPy is the acronym for the HEMA-UPy copolymers where HEMA is 2-hydroxyethyl methacrylate and UPy is ureidopyrimidinone) and prepare the epoxy composites is reported in the “Supplementary Materials” section.

**Figure 1:**
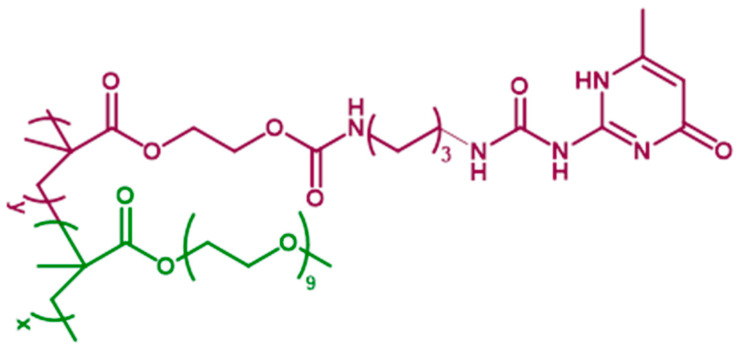
Chemical structure of PP-co-HUPy copolymer.

The HEMA-UPy copolymers (HUPy) were synthesized following a procedure already adopted in the literature [49,50]. Two different percentages of HUPy units were incorporated into the Poly-PEGMA polymer, 2.5 and 7.8% by weight, obtaining the samples PP-2.5-HUPy and PP-7.8-HUPy, respectively. Figure 1 shows the chemical structure of the PP-co-HUPy copolymer, evidencing the poly-PEGMA chains in green and the HEMA-UPy moiety in purple. 

The composition and preparation procedure of the host epoxy matrix, labeled in this paper with the acronym EP, are defined in the “Supplementary Materials” section.

The description of the preparation of UPy-Copolymers in the epoxy systems, namely EP-5PP-2.5-HUPy and EP-5PP-7.8-HUPy, is contained in the “Supplementary Materials” section.

### 2.2. Methods

In this paper, the epoxy-based samples were subjected to various characterization techniques whose exhaustive technical information is contained in the “Supplementary Materials” section. In particular, thermal analyses using Thermogravimetric Analyses (TGA) and Differential Scanning Calorimetry (DSC), structural investigations using Fourier transform infrared spectroscopy (FTIR), dynamic-mechanical analysis (DMA), and morphological investigation using Field Emission Scanning Electron Microscopy (FESEM) (mod. LEO 1525, Carl Zeiss SMT AG, Oberkochen, Germany) were carried out. The epoxy-based samples were etched [53] before the FESEM observation. 

## 3. Results

### 3.1. Thermal Analyses

**Figure 2:**
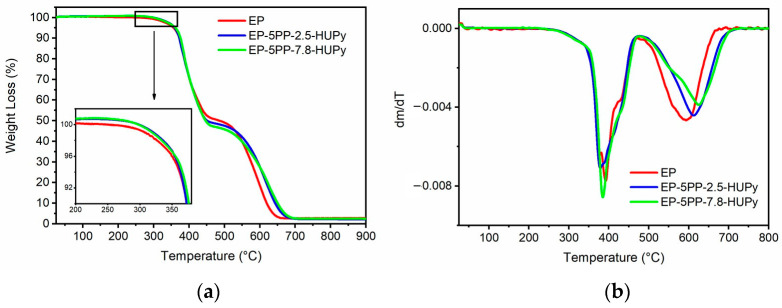
Profiles of (**a**) TGA (with inset focusing on the range of temperatures between 200 °C and 380 °C) and (**b**) DTG curves for the epoxy-based samples EP, EP-5PP-2.5-HUPy, and EP-5PP-7.8-HUPy.

The thermal behavior of the formulated epoxy samples was investigated by Thermogravimetric Analysis (TGA) and Differential Scanning Calorimetry (DSC). TGA measurements aimed to understand how the presence of PP-co-HUPy copolymers could influence the thermal resistance of the epoxy matrix. Figure 2 shows the profiles of (a) TGA (with the inset focusing on the range of temperatures between 200 °C and 380 °C) and (b) DTG (derivative thermogravimetry) curves for the epoxy-based samples EP, EP-5PP-2.5-HUPy, and EP-5PP-7.8-HUPy, while Table 1 displays the values of initial thermal degradation temperature (T_d5%_), defined as the temperature corresponding to a weight loss of 5 wt%, and the values of T_max1_ and T_max2_, representing the temperature at which the thermal degradation rate achieves the maximum value [54]. As evidenced by the inset of Figure 2a and the T_d5%_ values, the introduction of 5 wt% of PP-Co-UPy copolymers within the epoxy resin leads to a slight increase in the thermal stability of the matrix. In particular, a major increase was recorded for the sample loaded with the copolymer containing 7.8 wt% of the HUPy unit. Considering the trend of the data reported by the authors of Ref. [50], the epoxy resin phase determines the values of the thermal degradation. A slight increase in the thermal stability of the resin containing the supramolecular elastomer is most likely due to a slight increase in the DC of the epoxy systems containing the Poly(PEGMA-co-UPy) phase, as highlighted below. The thermal decomposition process shows two main steps, which substantially reflect the characteristic thermal profile belonging to the employed resin. The second stage reveals an increase in the thermal resistance for the EP-5PP-2.5-HUPy and EP-5PP-7.8-HUPy composites, which display values of T_max2_ 20 °C and 33 °C higher than the unfilled EP epoxy sample, respectively, as expected for systems characterized by a higher DC. Figure S1 in the “Supplementary Materials” section shows the comparison between (a) TGA and (b) DTG curves for the epoxy-based samples EP, EP-5PP-2.5-HUPy, and EP-5PP-7.8-HUPy (continuous curves) and the Poly(PEGMA)-based samples PP-2.5-HUPy and PP-7.8-HUPy (dashed curves).

A DSC investigation was used to evaluate the influence of the PP-co-HUPy copolymers on the curing degree (DC) of the epoxy resin EP. DC was evaluated using Equation (1), where Δ*H_Tot_* is the total heat of the reaction required to completely cure the samples and Δ*H_Res_* is the residual heat of the reaction of the partially cured samples [55].

(1) $$DC=\frac{{∆H}_{Tot}-{∆H}_{Res}}{{∆H}_{Tot}}\times 100$$

**Figure 3:**
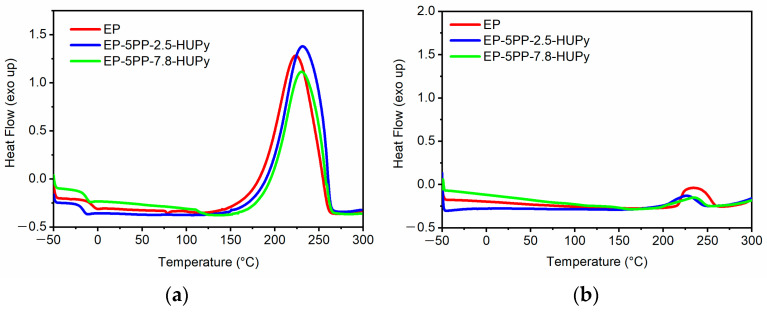
Thermograms of (**a**) uncured EP, EP-5PP-2.5-HUPy, and EP-5PP-7.8-HUPy samples and (**b**) partially cured EP, EP-5PP-2.5-HUPy, and EP-5PP-7.8-HUPy samples.

Table 2 illustrates the values of Δ*H_Tot_* and Δ*H_Res_*, which correspond to the areas under the thermograms of Figure 3.

In particular, the DSC analysis of the uncured samples was carried out using a dynamic heating program that contemplates three stages in the temperature range between −50 and 300 °C, namely, (a) the first run from −50 up to 300 °C with a scan rate of 10 °C min^−1^, (b) the second run from 300 to −50 °C with a scan rate of 50 °C min^−1^, and (c) the third run from −50 up to 300 °C with a scan rate of 10 °C min^−1^. These last scans are not reported here because no further curing reactions occur, as evident from the absence of the exotherm peak (see “third run” of all samples in Figure S2 in the “Supplementary Materials” section). The samples cured in the oven for 1 h at 125 °C and 3 h at 200 °C resulted in being not totally cured. However, they are characterized by a high curing degree that is usually suitable for practical applications. These last samples (cured with cycles performed through a sequence of two isothermal regimes) were then analyzed by a single heating run from −50 to 300 °C with a scan rate of 10 °C min^−1^. Considering the ${∆H}_{Res}$ of DSC curves illustrated in Figure 3b and applying Equation (1), the curing degree was found to range between 91.6 and 95.4, as shown in Table 2.

The results of DSC measurements highlight that the presence of the PP-Co-UPy copolymers leads to an increase in the curing degree of the epoxy matrix. The curing degree increases from 91.6% for the EP sample to 96.1% and 95.4% for EP-5PP-2.5-HUPy and EP-5PP-7.8-HUPy samples, respectively. The PP-Co-UPy copolymers act as agents promoting the curing reactions in the host matrix. It is most likely that the movement of the elastomer chains to reach a suitable arrangement for self-assembly in the fluid phase of the mixture also favors the cross-linking reactions, making the interactions between the epoxy precursor and the hardener agent easier. This determines a consequent increase in the curing degree while operating in the same thermal conditions as the EP epoxy matrix. 

### 3.2. FTIR Spectroscopy 

FTIR analysis was carried out to study the functional groups during the polymerization reactions. It is well known that after polymerization, the cross-linked epoxy matrix shows O-H functional groups [56]. 

**Figure 4:**
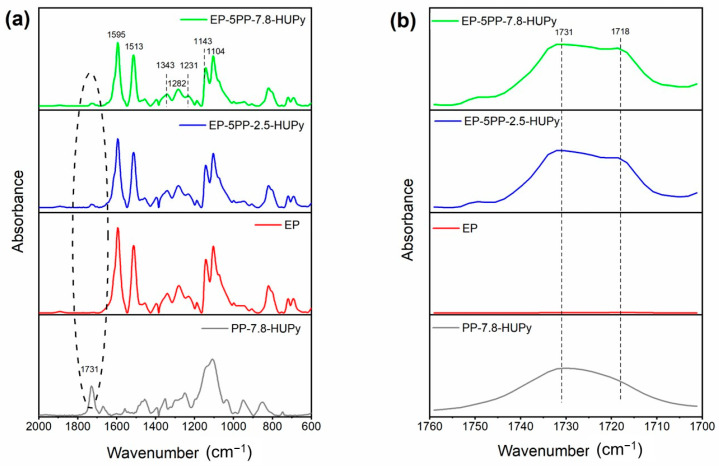
FTIR spectra of the samples EP-5PP-7.8-HUPy, EP-5PP-2.5-HUPy, EP, and PP-7.8-HUPy: (**a**) in the range of wavenumbers between 2000 and 600 cm^−1^; (**b**) enlargement in the range between 1760 and 1700 cm^−1^.

Figure 4 shows the FTIR spectra of the samples EP-5PP-7.8-HUPy, EP-5PP-2.5-HUPy, EP, and PP-7.8-HUPy: (a) in the range of wavenumbers between 2000 and 600 cm^−1^; (b) enlargement in the range between 1760 and 1700 cm^−1^. In particular, Figure 4a shows the FTIR spectrum of the PP-7.8-HUPy copolymer, compared to the FTIR spectra of the epoxy formulations EP, EP-5PP-2.5-HUPy, and EP-5PP-7.8-HUPy. Almost all signals detected in the spectra of the epoxy-based materials belong to the thermoset epoxy matrix, such as the signals at 1595 and 1513 cm^−1^, attributed to the stretching vibration of the benzene ring, the C–N stretching bands at 1343 cm^−1^ for tertiary aromatic amines and at 1282 cm^−1^ for aliphatic amines, the peak at 1231 cm^−1^ due to C–O stretching vibration of secondary alcohols, and the stretching vibration bands at 1143 and 1104 cm^−1^ attributed to sulfone group S=O of the curing agent [57,58,59,60]. The only signal belonging to the PP-Co-HUPy that can be detected in the spectra of the epoxy samples loaded with the copolymers is the band of the ester C=O carbonyl group at 1731 cm^−1^, evidenced by the dashed circle. Focusing our attention on this peak through the magnification between 1760 and 1700 cm^−1^ in Figure 4b, it can be observed that this signal shows a shoulder band at a lower wavenumber (1718 cm^−1^) that could be due to the presence of C=O groups involved in H-bond interactions [61,62] between the carbonyl group present on the surface of the elastomeric segregated crystals and the -OH groups of the crosslinked resin. This evidences that the elastomeric crystals, although segregated in a different phase, are well anchored to the hosting matrix. 

**Figure 5:**
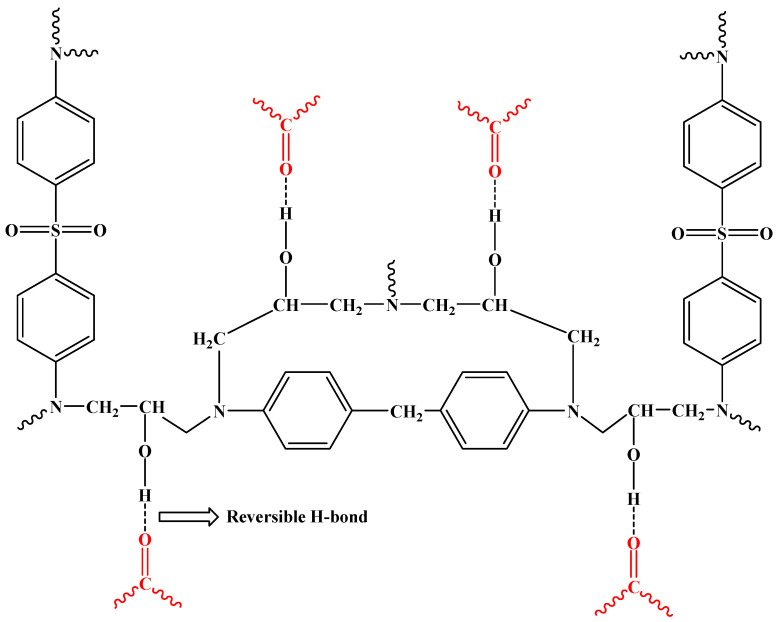
This picture illustrates a portion of crosslinked resin that interacts via H-bond with the carbonyl groups (red depicted) of the UPy-based copolymer.

Figure 5 illustrates a portion of crosslinked resin that interacts via H-bond with the carbonyl groups (depicted in red) of the UPy-based copolymer not involved in self-assembly interactions on the surface of the crystals. 

It is worth noting that, also in the spectrum of the pristine copolymer, the C=O shows a small shoulder band around 1718 cm^−1^ that becomes more evident when the PP-Co-HUPy copolymer is added to the epoxy matrix, thus indicating a different chemical environment able to influence the C=O groups involved in H-bond interactions. 

**Figure 6:**
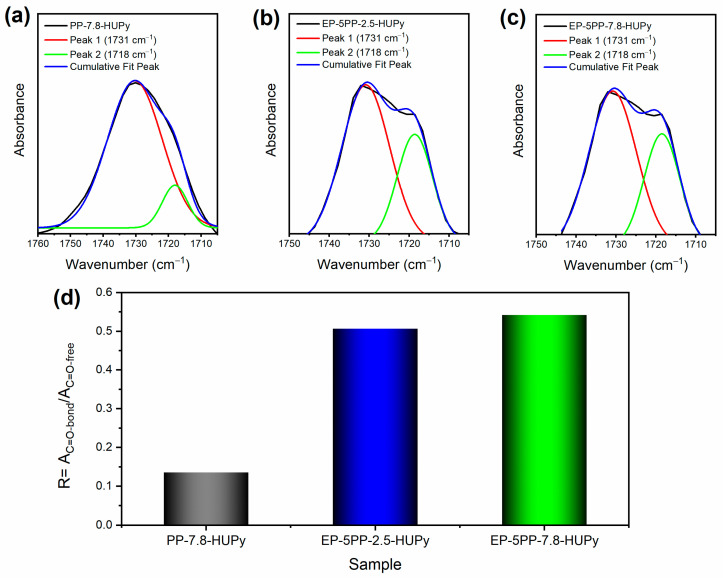
Results of the deconvolution procedure related to the region of the C=O group for the samples: (**a**) PP-7.8-HUPy; (**b**) EP-5PP-2.5-HUPy; (**c**) EP-5PP-7.8-HUPy; (**d**) values of the ratio R = A_C=O-bond_/A_C=O-free_ for the analyzed samples.

To better quantify this effect, the band of the C=O group has been decomposed into different components using a complex fitting in which a Gaussian contribution has been considered [56,61,63]. The results of this deconvolution procedure for the PP-7.8-HUPy and the epoxy samples are shown in Figure 6a–c. In particular, two different peaks have been considered for this deconvolution procedure. The peak at approximately 1718 cm^−1^ has been attributed to hydrogen bonding interactions, whereas the peak at the higher frequency, around 1731 cm^−1^, has been attributed to non-hydrogen bonded C=O groups. The ratio R, between the area of the C=O bonded signal (A_C=O-bond_) and the area of the free C=O signal (A_C=O-free_), has been considered to assess the extent of H-bonding interactions present in the materials.

The results of this evaluation are shown in the histogram of Figure 6d. The data highlight an increase in hydrogen bonds in the epoxy samples loaded with the copolymers; as for these materials, the ratio R = A_C=O-bond_/A_C=O-free_ shows slightly higher values. 

The spectra profiles, in the analyzed range of wavenumbers, confirm the good anchoring of the segregated crystals in the epoxy matrix. To verify this hypothesis, FESEM images of the samples, after a strong etching procedure, were analyzed. The results are presented and discussed in Section 3.4. 

### 3.3. Dynamic Mechanical Analyses

Dynamic mechanical analysis (DMA) was carried out to evaluate the mechanical behavior of the analyzed systems due to the presence of the copolymers within the host matrix. Through DMA, we obtained information on three main parameters, namely the storage modulus, loss modulus, and tan δ, which are used to describe the viscoelastic properties of materials. More precisely, the storage modulus is the measure of the material’s ability to store energy when it is deformed. It represents the elastic behavior of the material, indicating how much energy is stored in the material’s structure during deformation. A higher storage modulus means the material is more elastic and can recover its original shape after removing the stress. The loss modulus quantifies the amount of energy that is dissipated as heat when the material is deformed. It reflects the viscous behavior of the material, showing how much energy is lost due to internal friction within the material. A higher loss modulus indicates that the material has better damping properties, which is useful in applications where energy absorption is required. Tan δ (tan delta) is the ratio of the loss modulus to the storage modulus. It provides a measure of the damping characteristic of the material. When tan δ is less than 1, the material behaves more elastically; when it is greater than 1, it behaves more viscously. The peak of tan δ typically occurs at the glass transition temperature (Tg) of the material. These parameters are crucial for understanding how a material will perform under mechanical stress and can help design materials with desired mechanical properties for specific applications.

**Figure 7:**
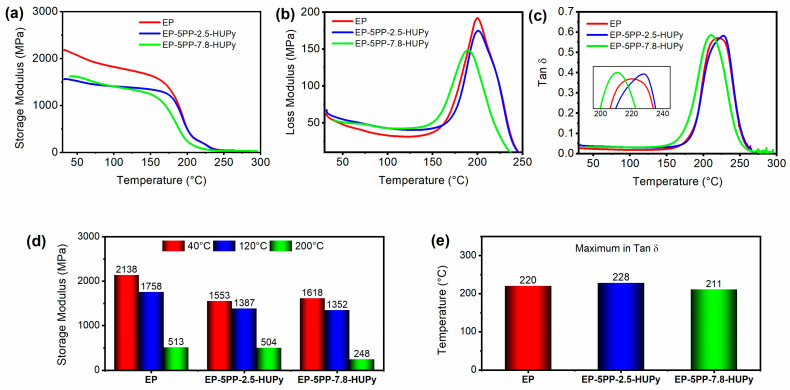
DMA results of the epoxy samples EP, EP-5PP-2.5-HUPy, and EP-5PP-7.8-HUPy: (**a**) storage modulus vs. temperature, (**b**) loss modulus vs. temperature, (**c**) tan δ vs. temperature (with inset focusing on the range of temperatures between 200 °C and 240 °C), (**d**) storage modulus at different temperatures, and (**e**) maximum Tan δ.

Figure 7 shows the DMA results of the epoxy samples EP, EP-5PP-2.5-HUPy, and EP-5PP-7.8-HUPy: (a) storage modulus vs. temperature, (b) loss modulus vs. temperature, (c) tan δ vs. temperature (with inset focusing on the range of temperatures between 200 °C and 240 °C), (d) storage modulus at different temperatures, and (e) maximum Tan δ.

The temperature range investigated is from 25 °C to 300 °C. From Figure 7a, we can observe that, compared to the EP epoxy matrix (red curve), for the EP-5PP-2.5-HUPy (blue curve) and EP-5PP-7.8-HUPy (green curve) samples, there is a general decrease in storage modulus values. This decrease is most likely attributable to a segregated phase of PP-co-HUPy copolymer crystals, as highlighted by FESEM images of the samples. In this regard, it is worth underlining that the copolymers in the uncured liquid EP epoxy mixture are completely soluble in both samples with different HUPy unit contents. Hence, phase separation occurs during the curing process realized through a curing cycle of 125 °C for 1 h, followed by 3 h at 200 °C (see section “Supplementary Materials”).

Strong, attractive interactions due to the hydrogen bonds established within the elastomeric phase can determine phase segregation. Since these interactions are directional in space, they are favored by an increase in temperature (favored by the high curing temperature), which allows segmental parts of the chains to better arrange themselves in a suitable way to establish the interactions sterically. The driving force is the lowering of the material’s energy, which is caused by attractive interactions.

For the three samples, EP, EP-5PP-2.5-HUPy, and EP-5PP-7.8-HUPy, the storage modulus values were measured at three different temperatures: 40, 120, and 200 °C, as shown in the histogram in Figure 7d, from which it is found that EP resin has the highest values of the storage modulus at all analyzed temperatures. In particular, for the EP sample, the storage modulus values are 2138 MPa at 40 °C, 1758 MPa at 120 °C, and 513 MPa at 200 °C. For the EP-5PP-2.5-HUPy sample, the storage modulus values are the following: 1553 MPa at 40 °C, 1387 MPa at 120 °C, and 504 MPa at 200 °C while for the EP-5PP-7.8-HUPy sample, the following storage modulus values were measured: 1618 MPa at 40 °C, 1352 MPa at 120 °C, and 248 MPa at 200 °C. The results indicate that, among the two samples loaded with the PP-co-HUPy copolymer, the one containing the highest percentage (7.8 wt%) of HUPy units at 40 °C shows a higher storage modulus value than that related to the sample loaded with the lowest percentage (2.5 wt%) of HUPy units, while a slight decrease in storage modulus is observed at 120 °C and then halved at the highest temperature of 200 °C. As regards the loss modulus, from Figure 7b, we can observe a somewhat similar trend for the unfilled EP resin and for the EP-5PP-2.5-HUPy sample while, instead, a decrease in the loss modulus is detectable for the sample EP-5PP-7.8-HUPy. The peak of tan δ, which typically occurs at the glass transition temperature (Tg) of the material, appears at a temperature value above 200 °C for all three formulations analyzed. In particular, from the histogram of Figure 7e, the values of maximum in Tan δ (Tg), representing the temperature at which the glass transition occurs, are 220, 228, and 211 for the epoxy formulations EP, EP-5PP-2.5-HUPy, and EP-5PP-7.8-HUPy, respectively. The three different samples manifest similar values in the glass transition temperature, proving that the epoxy phase influences this parameter. This result confirms consistent phase separation in the analyzed samples.

### 3.4. Field Emission Scanning Electron Microscopy (FESEM)

**Figure 8:**
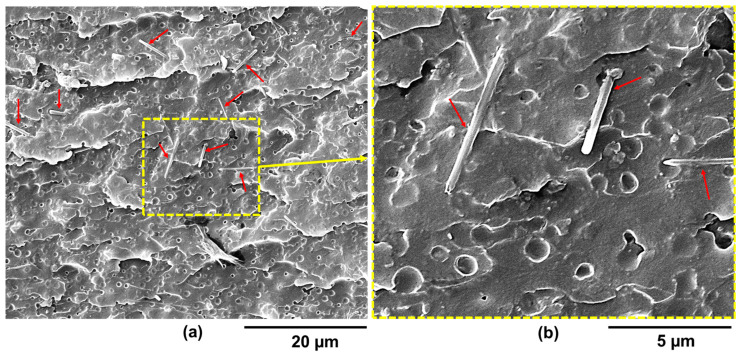
FESEM pictures of the epoxy sample EP-5PP-2.5-HUPy at two different magnifications: (**a**) scale bar of 20 µm and (**b**) scale bar of 5 µm. The red arrows indicate the presence of copolymer crystals with the shape of bars on the surface of the sample. The larger yellow box in the Figure 8b indicates the magnification corresponding to the area delimited by the smaller yellow box in the Figure 8a

**Figure 9:**
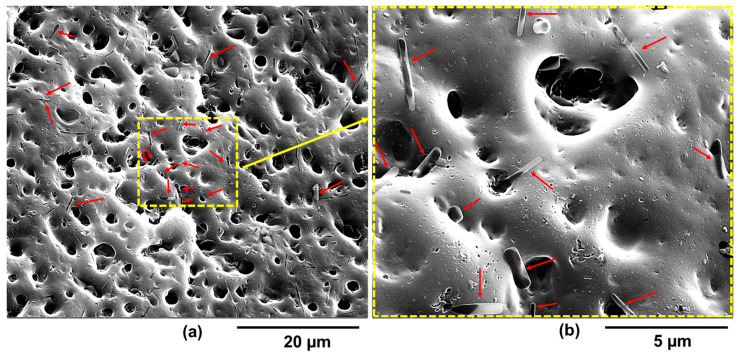
FESEM pictures of the epoxy sample EP-5PP-7.8-HUPy at two different magnifications: (**a**) scale bar of 20 µm and (**b**) scale bar of 5 µm. The red arrows indicate the presence of copolymer crystals with the shape of bars on the surface of the sample. The larger yellow box in the Figure 9b indicates the magnification corresponding to the area delimited by the smaller yellow box in the Figure 9a.

The FESEM morphological characterization carried out on the etched fracture surfaces of the epoxy samples with different contents of HUPy units proved to be crucial to understanding, at the microstructure level, why the presence of the PP-co-HUPy copolymer does not activate the self-repairing function. In this regard, for each of the two samples EP-5PP-2.5-HUPy and EP-5PP-7.8-HUPy, images at two different magnifications are shown in Figure 8 and Figure 9, respectively: (a) a scale bar of 20 µm and (b) a scale bar of 5 µm. The samples appear to have superficial cavities due to the strong etching chemical procedure. It is possible to observe the presence of copolymer crystals on the entire surface of the samples, which have the shape of bars, indicated by the red arrows (Figure 8a and Figure 9a). For a clearer and more effective visualization of the shape and size of the crystals as well as their distribution within the matrix, Figure 8b and Figure 9b show the images dotted in yellow at higher magnifications, which correspond to the areas of the surface of the two samples, which are delimited by the yellow dotted line in Figure 8b and Figure 9b at lower magnifications. The presence of crystals of the PP-co-HUPy copolymer, undoubtedly more numerous in the EP-5PP-7.8-HUPy sample containing a greater percentage of HUPy units, indicates phase segregation, which is responsible for the absence of the self-healing functionality in the systems.

It is clear that the supramolecular elastomer’s polymer chains cannot activate self-healing mechanisms within the epoxy matrix because they are only self-assembled among themselves, although anchored in the resin.

## 4. Conclusions

In this work, we have analyzed the possibility of transferring the self-healing ability of PP-co-HUPy copolymers to epoxy systems. UPy (ureido-pyrimidinone) is an interesting group capable of forming quadruple hydrogen bonds, which is a notable advancement considering that most hydrogen bonds consist of single to double bonds. The formulated epoxy samples EP-5PP-2.5-HUPy and EP-5PP-7.8-HUPy did not show self-healing efficiency due to the formation of phase segregation that originates during the curing process of the samples at high temperatures. Although the PP-co-HUPy copolymers are completely soluble in the fluid initial epoxy matrix EP, the presence of segregated crystals of PP-co-HUPy copolymers was directly detected by FESEM morphological analysis in the cured samples. This behavior is most likely a result of the strong, attractive interactions due to the hydrogen bonds established within the elastomeric phase. Furthermore, the crystals act as promoters of the curing degree (DC) of the hosting epoxy matrix. The increase in the degree of curing is most likely due to the greater mobility of the resin segments activated by the elastomeric chains that tend to assemble during the high-temperature cure reactions.

Dynamic mechanical analysis (DMA) shows that storage modulus values for epoxy systems containing PP-co-HUPy units are lower than those of the unfilled resin EP. This could be a direct consequence of the phase segregation, as also evidenced by the detection of similar values in the Tg. As a future perspective, the authors intend to compatibilize the epoxy precursor to avoid phase segregation and subsequently evaluate the effect of compatibilization on curing temperature on the possibility of transferring self-healing functionality to epoxy systems.

## Figures and Tables

**Table 1:** Thermogravimetric data of the epoxy-based samples EP, EP-5PP-2.5-HUPy, and EP-5PP-7.8-HUPy.

Samples	T_d5%_	T_max1_	T_max2_
EP	356.9	390.2	588.7
EP-5PP-2.5-HUPy	359.8	377.7	608.7
EP-5PP-7.8-HUPy	361.9	383.3	621.7

**Table 2:** Data on DSC analyses of the epoxy-based samples EP, EP-5PP-2.5-HUPy, and EP-5PP-7.8-HUPy.

Samples	Δ*H_Tot_* (J·g^−1^)	Δ*H_Res_* (J·g^−1^)	*DC* (%)
EP	499.0	42.0	91.6
EP-5PP-2.5-HUPy	520.3	20.4	96.1
EP-5PP-7.8-HUPy	414.0	19.2	95.4

## Data Availability

Data are contained within the article and Supplementary Materials.

## Supplementary Materials

The following supporting information can be downloaded at: https://www.mdpi.com/article/10.3390/polym16111509/s1, Experimental section: detailed information on materials and methods, Figure S1. Comparison between: (a) TGA and (b) DTG curves, for the epoxy-based samples EP, EP-5PP-2.5-HUPy, and EP-5PP-7.8-HUPy (continuous curves) and the Poly(PEGMA) based samples PP-2.5-HUPy and PP-7.8-HUPy (dashed curves), Figure S2. DSC analysis of the uncured samples: first run from −50 up to 300 °C with a scan rate of 10 °C min^−1^ (red curves); second run from 300 to −50 °C with a scan rate of 50 °C min^−1^ (blue curves); third run from −50 up to 300 °C with a scan rate of 10 °C min^−1^ (black curves).
