# Supplementary material for: Verification of the Self-Healing Ability of PP-co-HUPy Copolymers in Epoxy Systems
Source: Polymers (Basel). 2024 May 27;16(11):1509. doi: 10.3390/polym16111509 (PMC11174561; doi:10.3390/polym16111509)
Supplement: Supplementary file 1 [file polymers-16-01509-s001.zip › polymers-3020445-supplementary.pdf]

Supplementary electronic materials

Verification of the Self-healing Ability of PP-co-HUPy Copolymers in Epoxy systems

Elisa Calabrese [1], Marialuigia Raimondo [1,*], Andrea Sorrentino [2], Simona Russo [3], Pasquale Longo [3], Annaluisa Mariconda [4], Raffaele Longo [1] and Liberata Guadagno [1,*]

Department of Industrial Engineering, University of Salerno, Via Giovanni Paolo II, 132, 84084 Fisciano, Italy; elicalabrese@unisa.it (E.C.); rlongo@unisa.it (R.L.)

[2] Institute of Polymers, Composites and Biomaterials (IPCB-CNR), via Previati n. 1/E, 23900 Lecco, Italy; andrea.sorrentino@cnr.it

[3] Department of Chemistry and Biology, University of Salerno, Via Giovanni Paolo II, 132, 84084 Fisciano, Italy; sirusso@unisa.it (S.R.); plongo@unisa.it (P.L.)

[4] Department of Science, University of Basilicata, Viale dell'Ateneo Lucano, 10, 85100 Potenza, Italy; annaluisa.mariconda@unibas.it (A.M.)

* Correspondence: mraimondo@unisa.it (M.R.); lguadagno@unisa.it (L.G.)

Experimental section: detailed information on materials and methods

All reagents for the synthesis of PP-co-HUPy copolymers and for the preparation of the epoxy composites were bought by Merck or TCI Chemicals. 2-amino-4-hydroxy-6-methylpyrimidine (>98%, TCI), hexamethylene diisocyanate (>98%, TCI), polyethylene glycol monomethyl ether methacrylate stabilized with MEHQ (PEGMA, Mw = 500, TCI), dibutyltin dilaurate (>95%, TCI), and 2-2′-azobis(2- methylpropionitrile) (AIBN, 98%, Merck) were used as received, whereas 2-hydroxyethyl methacrylate stabilized with MEHQ (HEMA, >95%, TCI) was distilled before use. N,N-dimethylformamide (DMF) and chloroform solvents were used without further purification.

The host epoxy matrix, labelled in this paper with the acronym EP, was composed by the tetrafunctional epoxy precursor "tetraglycidyl methylene dianiline", the bifunctional reactive diluent "1,4-butanedioldiglycidylether" and the curing agent "4,4′-diaminodiphenyl sulfone". The preparation of the unfilled epoxy matrix EP involved the mixing of the precursor with the diluent, in a ratio 80/20, in order to obtain a liquid mixture in which the solid curing agent was completely solubilized (under magnetic stirring for 1 h at 120°C), in an amount of 55 phr with respect to the precursor. After the degassing procedure (in vacuum for 1 h at 100°C), the resulting liquid mixture was poured in a rectangular metallic mold and polymerized for 1 h at 125°C, followed by 3 h at 200°C, producing the cured epoxy sample EP.

The epoxy systems based on PP-co-HUPy copolymers, namely EP-5PP-2.5-HUPy and EP-5PP-7.8-HUPy, were prepared by adding 5wt% of PP-co-HUPy copolymer in the liquid uncured epoxy mixture, after the solubilization of the curing agent. In order to allow a good dispersion of the copolymer inside the epoxy matrix, the mixture was heated at 120 °C for 1 h and then it was sonicated (Hielscher model UP200S-24 kHz high power ultrasonic probe) for 20 minutes. The resulting blend was thermoset by following the curing cycle previously described.

The epoxy-based samples were thermally investigated by Thermogravimetric Analyses (TGA) and Differential Scanning Calorimetry (DSC). TGA was carried out on the cured samples by using a Mettler TGA/SDTA 851 analyzer, in air flow, in the temperature range between 25-900 °C and at heating rate of 10 °C min^{-1}. DSC was performed on uncured, totally cured and partially cured epoxy materials, by using Mettler DSC 822/400 (Mettler-Toledo Columbus, OH, USA) equipped with DSC cell purged with nitrogen and chilled with liquid nitrogen for sub-ambient measurements. The samples were scanned from -50 °C to 300 °C.

The FTIR spectra were obtained in absorbance by using a Bruker Vertex 70 FTIR-spectrophotometer with a resolution of 2 cm^{-1} (32 scans collected) in the range of 4000-400 cm^{-1}. The FT-IR spectrum of the uncured

epoxy-based formulation was collected by spreading the liquid mixture on the KBr pellet, while for the thermoset epoxy samples the spectra were obtained by dispersing powder of the samples in KBr pellets.

Dynamic mechanical analysis (DMA) was carried out on the epoxy samples through a DMA 2980 (TA instrument). Rectangular samples with dimensions 3×10×35mm^3 were tested by applying a variable flexural deformation in three points bending geometry. The displacement amplitude was set to 0.1% and the frequency to 1 Hz. These conditions were chosen to characterize the linear viscoelastic response of the materials and reduce the noise to signal ratio. The range of temperature analyzed was from 25°C to 300 °C at the constant scanning rate of 3°C min^{-1}. Before tests, calibration of clamp compliance, force and furnace temperature has been carried out.

Micrographs of the epoxy composites were obtained using FESEM (mod. LEO 1525, Carl Zeiss SMT AG, Oberkochen, Germany). All samples were placed on a carbon tab previously stuck to an aluminum stub and were covered with a 250 Å-thick gold film using a sputter coater (Agar mod. 108 A – Agar Scientific, Stansted, UK). They were etched before the observation by FESEM. The etching reagent was prepared by stirring 1.0 g potassium permanganate in a solution mixture of 95 mL sulfuric acid (95–97%) and 48 mL orthophosphoric acid (85%). The filled resins were immersed into the fresh etching reagent at room temperature and held under agitation for 5 h. Subsequent washings were done using a cold mixture of two parts by volume of concentrated sulfuric acid and seven parts of water. Afterward the samples were washed again with 30% aqueous hydrogen peroxide to remove any manganese dioxide. The samples were finally washed with distilled water and kept under vacuum for 5 days before being subjected to morphological analysis.

Figure S1 shows the comparison between: (a) TGA and (b) DTG curves, for the epoxy-based samples EP, EP-5PP-2.5-HUPy, and EP-5PP-7.8-HUPy (continuous curves) and the Poly(PEGMA)-based samples PP-2.5-HUPy, and PP-7.8-HUPy (dashed curves).

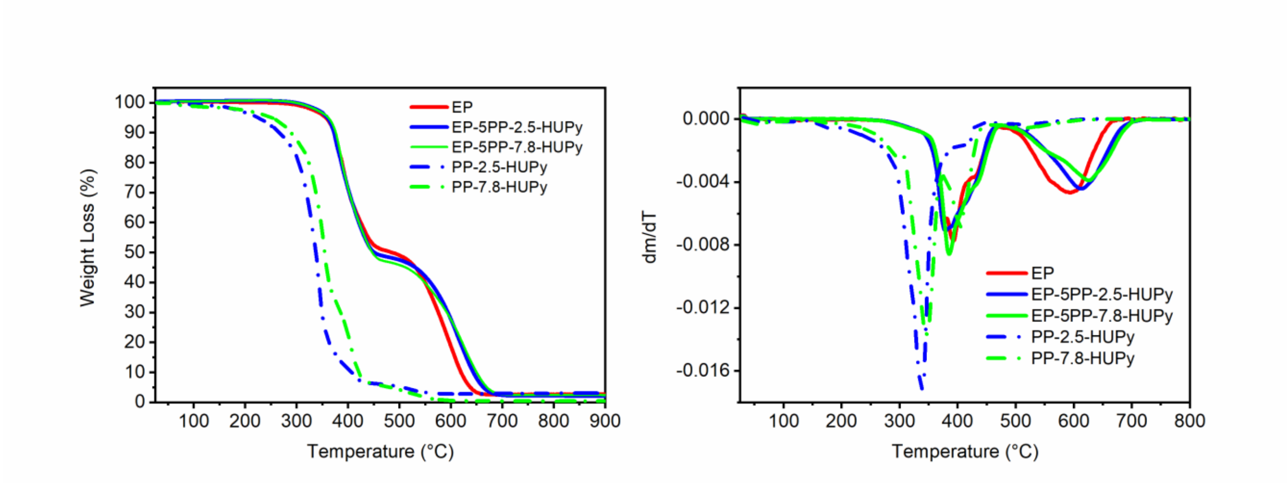

(**a**) (**b**)

Figure S1. Comparison between: (a) TGA and (b) DTG curves, for the epoxy-based samples EP, EP-5PP-2.5-HUPy, and EP-5PP-7.8-HUPy (continuous curves) and the Poly(PEGMA) based samples PP-2.5-HUPy, and PP-7.8-HUPy (dashed curves).

Figure S2 shows the DSC curves of the uncured samples. In particular, three runs have been shown for all samples: the first run from -50 up to 300 °C with a scan rate of 10 °C min^{-1} (red curves); the second run from 300 to -50 °C with a scan rate of 50 °C min^{-1} (blue curves) and the third run from -50 up to 300 °C with a scan rate of 10 °C min^{-1} (black curves).

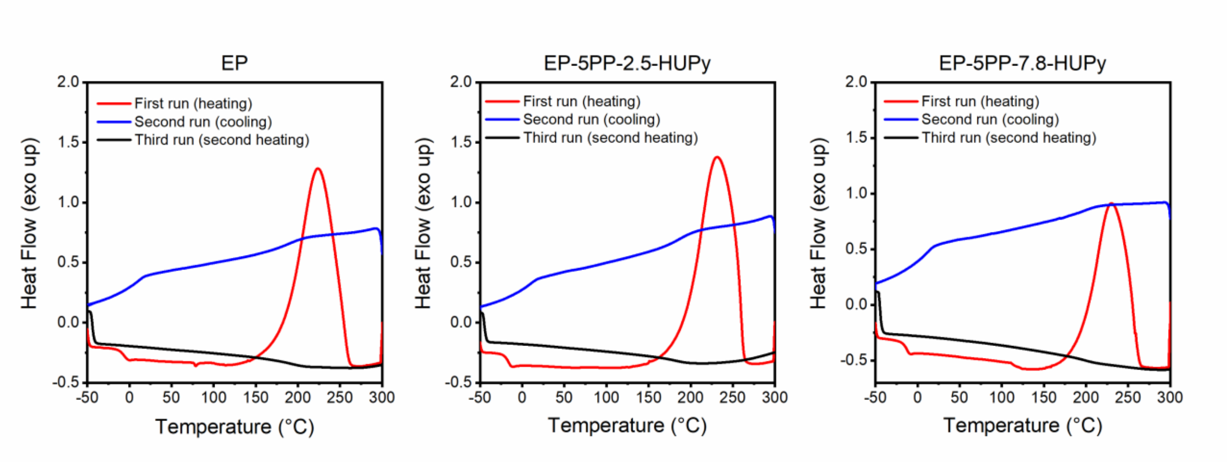

Figure S2. DSC analysis of the uncured samples: first run from -50 up to 300 °C with a scan rate of 10 °C min^{-1} (red curves); second run from 300 to -50 °C with a scan rate of 50 °C min^{-1} (blue curves); third run from -50 up to 300 °C with a scan rate of 10 °C min^{-1} (black curves).

Figure S2 clearly shows that the black curves do not present polymerization isotherms. Hence, after the dynamic cycle, the samples are totally cured.
